# Efficacy of DA-5204 (Stillen 2X) for patients with gastroesophageal reflux disease: A randomized, double-blind, placebo-controlled pilot study

**DOI:** 10.1097/MD.0000000000022729

**Published:** 2020-10-30

**Authors:** Jae Ho Cho, Hyuk Yoon, Cheol Min Shin, Young Soo Park, Nayoung Kim, Dong Ho Lee

**Affiliations:** Department of Internal Medicine, Seoul National University Bundang Hospital, Seongnam, Republic of Korea.

**Keywords:** *artemisia asiatica*, gastroesophageal reflux disease, proton pump inhibitor, reflux esophagitis

## Abstract

**Background/Aim:**

Proton pump inhibitor (PPI) alone is not satisfactory for the treatment of gastroesophageal reflux disease (GERD). Therefore, we investigated the efficacy of DA-5204 (Stillen 2X, 90 mg of *Artemisia asiatica* 95% ethanol extract per tablet) and PPI combination therapy on GERD in comparison to PPI alone.

**Methods:**

This randomized, double-blind, placebo-controlled study randomly assigned 70 patients with endoscopically proven esophageal mucosal injury (Los Angeles classification grade A or B) into 2 groups: pantoprazole 40 mg once daily with DA-5204 twice daily (DA-5204 group) or pantoprazole 40 mg once daily with placebo twice daily (placebo group) for 4 weeks. The primary endpoint was endoscopic healing rate. The secondary endpoint was sufficient relief (≥50% reduction) of symptoms using GERD Questionnaire.

**Results:**

Final analyses included 29 patients with the DA-5204 group and 30 patients with the placebo group. At weeks 4, there was no significant difference in the endoscopic healing rate between the 2 groups (DA-5204 vs placebo; 96.6% vs 93.3%; *P* = 1.000). However, the rate of residual minimal change was significantly lower in the DA-5204 group (5/28, 17.9%) than in the placebo group (17/28, 60.7%) (*P* *<* .001). The rates of symptom relief were not different between the DA-5204 group and the placebo group (all *P* > .05).

**Conclusion:**

Combined therapy with PPI and DA-5204 has no additional effect on the endoscopic healing rate compared to PPI alone. However, it may be beneficial in resolving minimal change.

## 1 Introduction

Gastroesophageal reflux disease (GERD) is a condition in which reflux of stomach contents into the esophagus causes troublesome symptoms, such as heartburn and acid regurgitation, and/or complications.^[1,2]^ GERD can be categorized into 2 types: erosive esophagitis is characterized by endoscopic evidence of mucosal injury such as erosion; and non-erosive reflux disease (NERD) is characterized by the presence of the symptoms of GERD without visible erosive changes on endoscopy.^[3–5]^ Untreated GERD can reduce quality of life and lead to complications, including esophageal ulcer, stricture, hemorrhage and Barrett's esophagus.^[6–8]^

Systematic reviews found the prevalence of GERD to be 10% to 30% in the Western countries,^[9,10]^ while lower in the Asia with less than 10% including South Korea.^[11–13]^ However, the prevalence of GERD in Eastern Asia has increased in recent years. This may be a consequence of aging of the population, westernization of the lifestyle and dietary habits, obesity, and decrease of the *Helicobacter pylori* infection.^[11,14,15]^

Proton pump inhibitors (PPIs) are the agents recommended as the first-line treatment for GERD.^[1,2]^ However, approximately 20% to 30% of patients with GERD have insufficient effect on PPI treatment.^[16–21]^ Thus, the development of a novel therapeutic strategy is awaited to control of remnant reflux symptoms despite taking PPI.

DA-5204 (Stillen 2X; Dong-A ST Co., Seoul, Korea) tablet, with 90 mg of *Artemisia asiatica* 95% ethanol extract per tablet, is a new formulation with longer intragastric retention of the active ingredient that improves DA-9601 (Stillen; Dong-A ST Co.). It has been administered to treat gastritis and gastric ulcers with antioxidative and cytoprotective actions on gastric mucosal damage.^[22–27]^ Previous reports were studies of gastric mucosal healing, while there was no data of the effect of DA-5204 on erosive esophagitis in human.

From this background, we evaluated the efficacy of DA-5204 and PPI combination therapy in patients with endoscopically confirmed GERD in comparison to PPI alone.

## 2 Methods

### 2.1 Study subjects

This randomized, single center, double-blind, placebo-controlled pilot study was conducted in Korea from June 2016 to December 2018. Patients were recruited from Seoul National University Bundang Hospital.

Inclusion criteria were as follows:

(1) patients aged 20 to 75 years and
(2) those with endoscopically proven esophageal mucosal injury (Los Angeles [LA] classification grade A or B).

Exclusion criteria were as follows:

(1) patients with an endoscopic finding of esophageal stricture, esophageal varix, Barrett's esophagus, peptic ulcer, gastrointestinal bleeding, Zollinger–Ellison syndrome or malignancy;
(2) patients who had undergone a previous gastrointestinal operation, such as an operation to inhibit gastric acid secretion, esophagectomy or gastrectomy (simple stomach perforation operation was excluded);
(3) patient who used any prokinetics, histamine-2 receptor antagonists, PPI, anticholinergic drugs (muscarinic receptor antagonists), gastrin receptor antagonists, protective factor enhancers, gastric mucosal protective agents or NSAIDs within 4 weeks of the screening test;
(4) women who were pregnant or lactating;
(5) women of childbearing age not using contraception; and
(6) patients with significant impairments in the hematologic, renal, cardiac, pulmonary, hematopoietic, and endocrine systems.

### 2.2 Study Protocol

**Figure 1:**
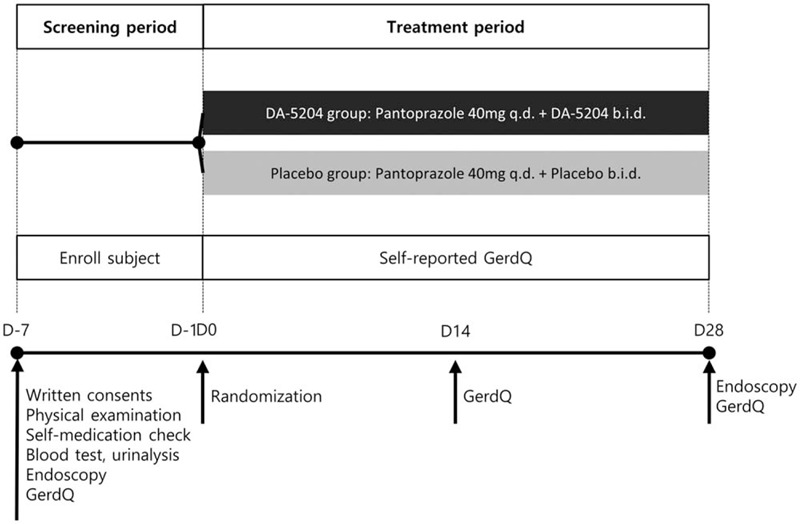
Overview of the study design. *q.d.* Once daily, *b.i.d.* twice daily, GerdQ, Gastroesophageal Reflux Disease Questionnaire.

Subjects who participated in the clinical study were received to blood tests, urinalysis, upper gastrointestinal endoscopy and rapid urease test for screening tests. The patients eligible for the screening test completed the Gastroesophageal Reflux Disease Questionnaire (GerdQ) to evaluate the symptoms of reflux esophagitis.^[28]^ The GerdQ is a self-administered questionnaire consisting of 6 questions (1, heartburn; 2, regurgitation; 3, epigastric pain; 4, nausea; 5, sleep disturbance due to heartburn or regurgitation; 6, use of over-the-counter medication to manage heartburn or regurgitation). Questions 1, 2, 5, and 6 are positive predictors of GERD, and questions 3 and 4 are negative predictors. Scores ranging from 0 to 3 indicate symptom frequency per week of positive predictors (0, no symptoms; 1, 1 day; 2, 2–3 days; 3, 4–7 days). The scores are used in reverse for negative predictors. And, all the patients were randomized (1:1 ratio) to the test group (DA-5204; Dong-A ST Co.) or the control group (placebo). The random allocation table was generated by a computer program. This study was conducted in a double-blind manner. Participants received either pantoprazole 40 mg once daily with DA-5204 twice daily or pantoprazole 40 mg once daily with placebo twice daily for 4 weeks. Patients were instructed by telephone to fill out the GerdQ for mid-term evaluation of symptoms 2 weeks after beginning the medication. Patients visited the clinical study center to fill out the GerdQ and received follow-up endoscopy 4 weeks after treatment. Compliance was determined by the number of remaining tablets per drug type at the follow-up visit. Data of patients with < 80% drug compliance were excluded in the per-protocol outcome analysis. The study design is shown in Fig. 1.

### 2.3 Efficacy assessments

The efficacy of the treatments was assessed by evaluating the change of esophageal mucosal injury on endoscopy and the relief of symptoms using the GerdQ.

### 2.4 Primary endpoint

The primary endpoint was the improvement of esophageal mucosal injury as assessed by endoscopy. Endoscopic score was defined as ranged from 0 to 3 (0, no visible mucosal injury, that is, LA grade N [normal]; 1, LA grade M [minimal change]; 2, LA grade A; 3, LA grade B).^[29–32]^ The healing rate on endoscopy was defined as the percentage of patients who had an endoscopic score of 0 (normal) or 1 (minimal change) at the follow-up endoscopy 4 weeks after treatment. Additionally, subgroup analysis was performed to differentiate subtle treatment response. The rate of minimal change was defined as the percentage of patients remaining in minimal change (score of 1) among healed patients (score of 0 or 1).

### 2.5 Secondary endpoint

The secondary endpoint was the improvement of reflux symptom using the GerdQ after 4 weeks of treatment. The subgroup analysis was performed with ≥8 score of the GerdQ; the cutoff value of 8 was found to have the highest sensitivity and specificity for the diagnosis of GERD.^[28,33]^ The sufficient relief of reflux symptom was defined as ≥50% reduction from the initial sum of scores for questions 1, 2, 5, and 6.

### 2.6 Safety assessments

Safety assessments included adverse events (AEs) and adverse drug reactions (ADRs), including any gastrointestinal symptoms and abnormalities in laboratory findings or vital signs. Complaint questionnaires were administered to assess for any harmful or untoward reactions experienced by a patient.

### 2.7 Sample size and statistical analysis

This study was a pilot study before full-scale verification and planned to test 30 patients per group, the recommended minimum number of subjects in the pilot study.^[34,35]^ Therefore, we recruited a total of 70 patients for each group of 35 subjects assuming a 15% drop-out rate.

Repeated measures analysis of variance was used to evaluate differences in efficacy of reflux symptoms associated with drug treatments between the 2 groups. The efficacy, such as sufficient relief of symptoms, healing rate and rate of minimal change on endoscopy, was analyzed by the *χ*^2^ or Fisher exact test. Inter-group comparisons of the other variables were conducted using the student *t*-test for continuous data and the *χ*^2^ or Fisher exact test for categorical data. *P*-value <.050 was defined as statistically significant difference. All statistical analyses were performed using SPSS, version 25.0 (IBM Inc., Chicago, IL).

### 2.8 Ethics statement

This study was reviewed by the Institutional Review Board of Seoul National University Bundang Hospital (B-1508-311-003). All procedures performed in this study involving human participants were in accordance with the 1964 Declaration of Helsinki and its later amendments or comparable ethical standards. Informed consent was submitted by all subjects when they were enrolled. This study was registered as a standard, randomized clinical trial (ClinicalTrials.gov: NCT03998969).

## 3 Results

### 3.1 Allocation of the patients

**Figure 2:**
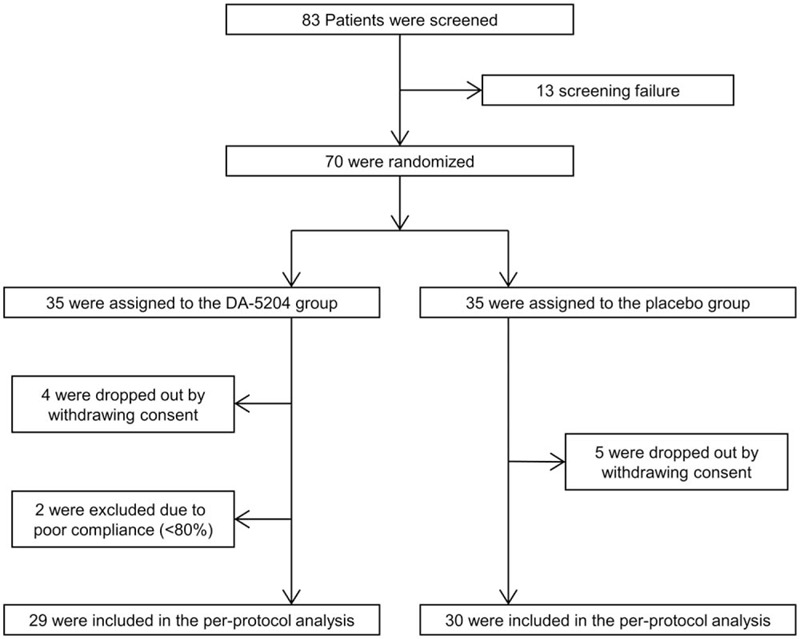
Flow diagram of study patients.

A total of 83 patients signed informed consent and participated in the screening. Of these, 70 patients who met the inclusion criteria were randomly assigned to the DA-5204 group or the placebo group. Out of the 9 patients who withdrew consent during the study, 61 patients finally completed the study. Except for the 2 patients with low drug compliance (<80%), consequently, 59 patients (*n* = 29 in the DA-5204 group and *n* = 30 in the placebo group) were available for the per protocol analysis. Fig. 2 presents the flow of study patients.

### 3.2 Demographic characteristics

Table 1 shows patients’ demographic and baseline characteristics. There were no differences between the 2 groups in terms of age, sex, height, weight, body mass index or smoking status, except for alcohol consumption. The other clinical factors, including LA classification grades, status of *Helicobacter pylori* infection, GerdQ scores prior to treatment or drug compliance, were also not significantly different between groups.

**Table 1:**
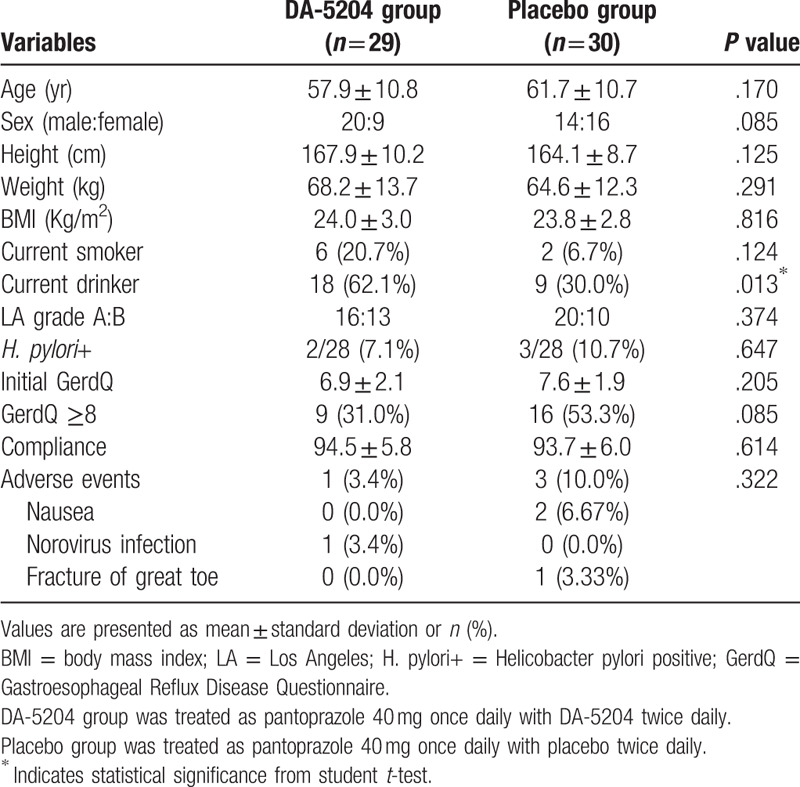
Baseline characteristics of the study population.

### 3.3 Primary efficacy endpoints

**Figure 3:**
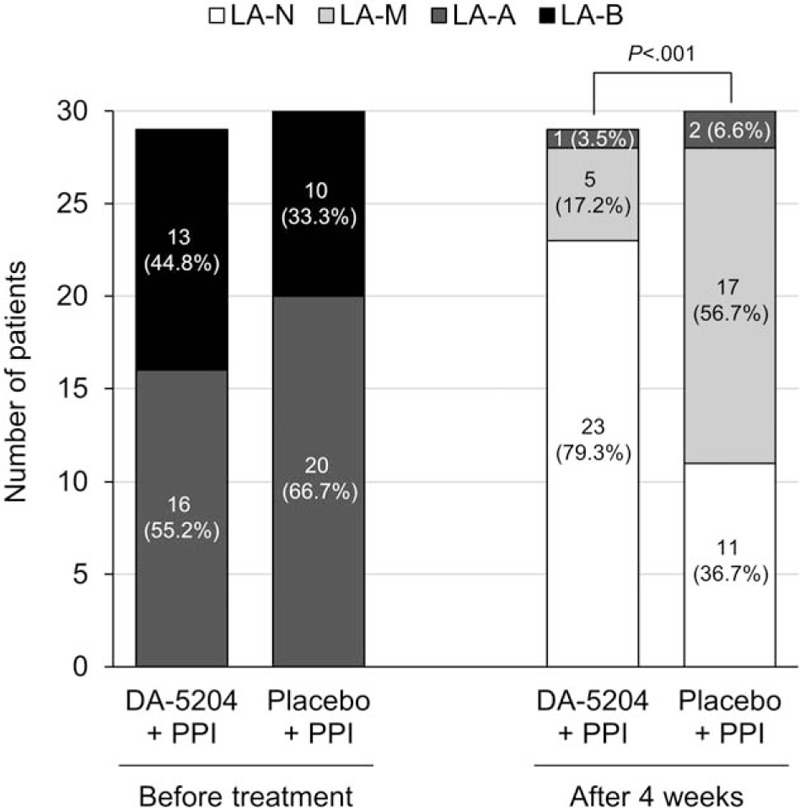
The distribution of Los Angeles classification grades before and after treatment in both the DA-5204 and placebo group. LA = Los Angeles; PPI = proton pump inhibitor.

**Figure 4:**
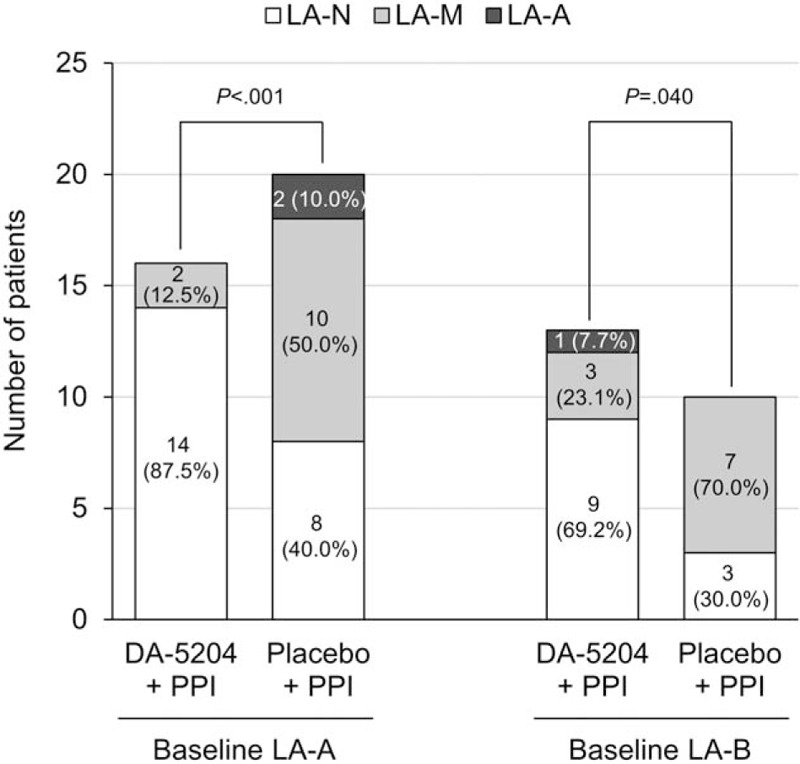
The distribution of Los Angeles classification grades after treatment by pre-treatment grades. LA = Los Angeles; PPI = proton pump inhibitor.

Prior to treatment, 16 of the 29 patients (55.2%) in the DA-5204 group and 20 of the 30 patients (66.7%) in the placebo group had LA grade A, and 13 of the 29 patients (44.8%) in the DA-5204 group and 10 of the 30 patients (33.3%) in the placebo group had LA grade B (*P* = .374). After 4 weeks of treatment, 23 patients (79.3%) were classified as grade N, 5 patients (17.2%) as grade M, and 1 patient (3.5%) as grade A in the DA-5204 group. In the placebo group, 11 patients (36.7%) were classified as grade N, 17 patients (56.7%) as grade M, and 2 patients (6.6%) as grade A (*P* < .001) (Fig. 3). Similar treatment responses were observed when the pre-treatment grades were divided into A and B. After 4 weeks of treatment for patients with pre-treatment LA grade A esophagitis, 14 patients (87.5%) improved as grade N and 2 patients (12.5%) as grade M in the DA-5204 group. In the placebo group, 8 patients (40.0%) improved as grade N and 10 patients (50.0%) as grade M, but 2 patients (10.0%) showed no change in the grade (*P* < .001). After treatment for patients with pre-treatment LA grade B esophagitis, 9 patients (69.2%) improved as grade N, 3 patients (23.1%) as grade M, and 1 patient (7.7%) as grade A in the DA-5204 group. In the placebo group, 3 patients (30.0%) improved as grade N and 10 patients (70.0%) as grade M (*P* = .040) (Fig. 4).

Table 2 shows the rates of healing after treatment. The healing rate was observed in 96.6% (28/29, 95% confidence interval [CI] 93.2–100.0%) of patients in the DA-5204 group and 93.3% (28/30, 95% CI 88.7–97.9%) of patients in the placebo group after 4 weeks of the treatment (*P* = 1.000). Subgroup analysis was performed on these healed patients to analyze the differences in subtle endoscopic changes. The rate of minimal change was observed in 17.9% (5/28, 95% CI 10.5–25.3%) of patients in the DA-5204 group and 60.7% (17/28, 95% CI 51.3–70.1%) of patients in the placebo group after treatment (*P* < .001). To summarize, the healing rate did not show a significant difference between the 2 groups after treatment. However, the rate of minimal change remaining after treatment among these healed patients was lower in the DA-5204 group than in the placebo group.

**Table 2:**
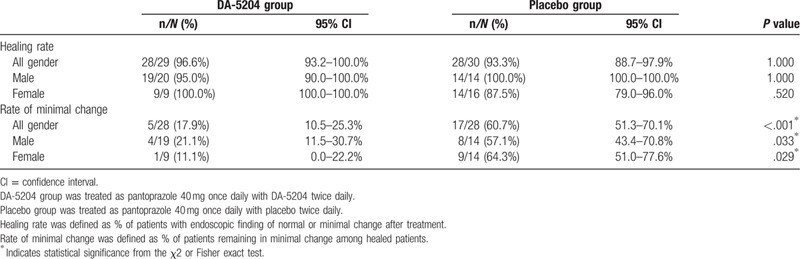
The efficacy of the treatment of esophageal mucosal injury on endoscopy between the 2 groups.

### 3.4 Secondary efficacy endpoint

**Figure 5:**
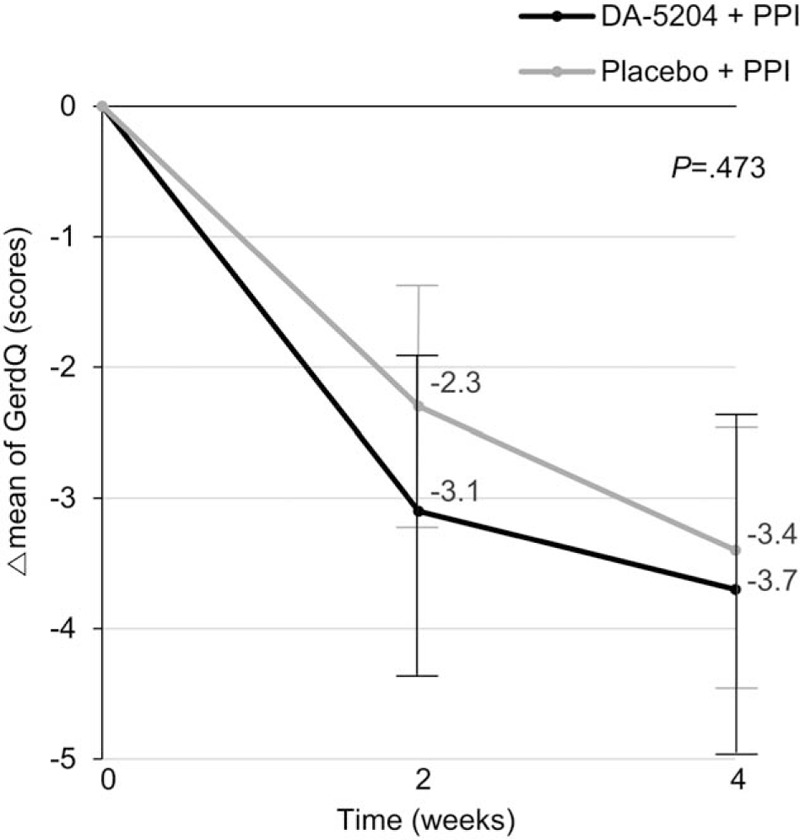
The delta changes of the GerdQ after treatment in the subgroup for ≥8 score of the GerdQ. The *P-*value was determined by the RM-ANOVA. GerdQ, Gastroesophageal Reflux Disease Questionnaire. RM-ANOVA = repeated measures analysis of variance.

We performed subgroup analysis for ≥8 score of the GerdQ. Delta means of the GerdQ scores were -3.1 ± 2.1 and -3.7 ± 1.8 in the DA-5204 group and -2.3 ± 1.7 and -3.4 ± 2.2 in the placebo group at 2 and 4 weeks, (*P* = .473), respectively (Fig. 5). There was no statistically significant difference between the 2 groups, although the scores of the GerdQ decreased in both groups after treatment. Table 3 shows sufficient relief of reflux symptom using GerdQ between the 2 groups. There was no significant difference between the 2 groups, according to subgroup of GerdQ or sex.

**Table 3:**
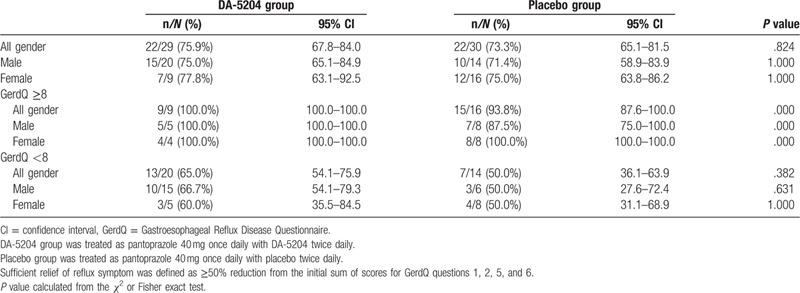
The efficacy of the sufficient relief of reflux symptoms using GerdQ between the 2 groups.

### 3.5 Safety

During the study period, AEs were reported in 1 patient (3.4%) in the DA-5204 group and 3 patients (10.0%) in the placebo group, and were not statistically significantly different between the 2 groups (Table 1). All those reported AEs were confirmed not to be ADR. No serious AE or ADR was reported.

## 4 Discussion

This was the first study assessing the effectiveness and safety of DA-5204 for patients with GERD. Following 4 weeks of the treatment, the overall healing rates of erosive esophagitis on endoscopy were not different between the combination therapy of pantoprazole and DA-5204 and the combination therapy of pantoprazole with placebo (96.6% *vs*. 93.3%; *P* = 1.000). This result was consistent with the treatment response of previous studies. One of these literatures was reported that standard dose PPI therapy had been found to have healing rates of 63% to 92% in Western patients with severe reflux esophagitis (LA grade C or D).^[36]^ Cho et al reported a complete healing rate of 83.9% after 4 weeks of treatment with pantoprazole 40 mg, assessed by endoscopy. They recruited patients with erosive esophagitis (LA grade A, 33; LA grade B, 27; LA grade C, 2) similar to our study.^[37]^ However, our study showed an interesting finding in addition to these results. Among endoscopically healed patients after 4 weeks of treatment, the proportion of LA grade N (normal) was higher than grade M (minimal change) in the DA-5204 group compared to the placebo group (82.1% *vs*. 39.3%; *P* < .001).

The reflux symptom, using GerdQ, improved in both DA-5204 (75.9%) and placebo groups (73.3%). However, there was no statistically significant difference between the 2 groups. In the subgroup analysis, patients with GerdQ ≥8, a valid score of being diagnosed as GERD, were observed to have sufficient relief of reflux symptom both the 2 groups, except 1 patient in the placebo group (100.0% and 93.8%). While patients with GerdQ < 8 were observed to have low sufficient relief of reflux symptom both the 2 groups (65.0% and 50.0%), because the initial symptoms of these patients were not definite, the assessment of symptomatic improvement would not have been satisfactory.

DA-5204, a phytopharmaceutical medicine derived from *Artemisia asiatica*, is a new formulation administered twice per day with a prolonged gastric retention time of the active ingredient instead of 3 times per day of DA-9601 and has demonstrated to be not inferior to DA-9601 for treating erosive gastritis.^[38]^ DA-9601, the predecessor of DA-5204, has been reported to have antioxidative and cytoprotective actions in various models of gastric mucosal damage.^[25–27]^ Eupatilin, a major component of DA-5204, has been shown to inhibit FeSO_4_-induced reactive oxygen species production and reduce oxidative-driven gene expression, resulting in the prevention of H_2_O_2_-induced gastric epithelial damage^[39]^ and the production of tumor necrosis factor (TNF)-α through modulation of p38 kinase and nuclear factor (NF)-κB-dependent pathways.^[40]^ Cytoprotective effects of DA-9601 have been reported in several animal and human studies. DA-9601 reduced the alcohol-induced hemorrhagic injury to the gastric mucosa in rats by inhibiting alcohol-induced xanthine oxidase^[41]^ and treated gastric mucosa in patients with erosive gastritis.^[23]^ These mucosal healing mechanisms are expected to be applied similarly to erosive esophagitis.

Adverse reactions were observed in 3.4% (1/29) of the patients with DA-5204 group and 10.0% (3/30) in the placebo group. In both groups, the main adverse reactions were nausea (2/59, 3.4%). No serious adverse reactions were observed.

This study had some limitations. First, the number of the study population was small. Second, we only enrolled patients with mild erosive esophagitis (LA grade A or B), not severe disease. We anticipated that DA-5204 add-on therapy to PPI would be more effective in severe esophagitis due to its mucosal healing effects. However, patients with erosive esophagitis in Asia were known to be milder in endoscopic severity, mostly (90%) LA grade A or B, than in the West.^[42–44]^ In addition, Korean data similarly reported that 90% to 100% of patients with erosive esophagitis were LA grade A or B.^[11]^ This study is valuable because it is clinical data from these Asian population that is consistent with our participants with mild esophagitis. Third, minimal change esophagitis has a bias due to the interobserver variation. To reduce this concern, in our study, 2 endoscopists independently reviewed the participants’ endoscopic images. Fourth, it is still controversial that the clinical implications of minimal change esophagitis are unclear. However, in Asia, as much as 50% to 70% of GERD patients had been found to be have NERD instead of erosive esophagitis.^[42]^ Especially in Japan, the majority of GERD seems to be NERD.^[32,44]^ Therefore, Japanese proposed a modified LA classification, including the minimal change as grade M and the esophagus without any mucosal change as grade N, so that NERD could be divided in more detail.^[31]^ One Japanese multicenter study reported that the frequency of abnormal acid reflux (% time pH <4 above 4%) was higher in patients with minimal change lesions than in patients without such changes (57.1% vs 11.8%), supporting a pathologic role of acid in the formation of minimal change esophagitis.^[45]^ One Korean study reported that the frequency of minimal change was higher in the patients with NERD than in the healthy control (71.9% vs 45.2%).^[46]^ In addition, studies with high-resolution magnifying endoscopes provide possible evidence. Kiesslich et al showed that minimal changes observed in high-resolution endoscope were more often in the patients with NERD than in the control group (27/39 vs 8/39, *P* = .005), histological abnormalities such as elongated papillae and basal cell hyperplasia were more common in the patients with NERD, and some of these endoscopic and histologic changes were improved after 4 weeks of treatment with esomeprazole 20 mg (*P* < .05).^[47]^ Edebo *et al.* reported that triangular indentations, apical mucosal breaks, and pinpoint blood vessels at distal esophagus were identified more frequently in the patients with NERD (*P* < .05).^[48]^ These studies suggest that minimal change may be related to the pathogenesis of GERD. Fifth, this study enrolled a large proportion of patients with asymptomatic esophagitis, making it difficult to show whether reflux symptoms were improved. Because patients with erosive esophagitis confirmed by endoscopy were preferentially recruited, and then the GerdQ questionnaire was completed, therefore, some of the patients with low scores of GerdQ were included (GerdQ <8; 20/29, 69.0% in the DA-5204 group and 14/30, 46.7% in the placebo group).

Meanwhile, dilated intercellular spaces (DIS) of esophageal mucosa, measured by electron microscopy, are frequently observed in patients with NERD and in patients with erosive esophagitis. In patients with GERD, DIS is regarded as a histological marker of epithelial damage induced by abnormal acid exposure, and is proposed as the missing link in the pathogenesis of symptoms in NERD.^[49]^ Furthermore, a small animal study published only in abstract form showed that antioxidants, such as vitamin C and N -acetylcysteine, may prevent DIS in esophageal epithelium that is exposed to weakly acidic solutions.^[50]^ Likewise, we believe that DA-5204 with antioxidant activity can be expected to have such a healing effect on DIS. From this perspective, large-scale studies on the recovery effects of cytoprotective agents for DIS in patients with NERD are necessary.

Nonetheless, the significance of this prospective study is that it is the first human data to evaluate the therapeutic response to DA-5204 on GERD. Our findings showed the possibility of an additional treatment option for DA-5204 as a combination with PPI for treating GERD.

In conclusion, DA-5204 did not affect the overall healing rate compared to placebo. However, it may be suggested that combined therapy with PPI and DA-5204 had a possible effect in healing subtle mucosal damage expressed by minimal change. Large-scale multicenter studies are warranted in the future.

## Author Contributions

Dong Ho Lee conceptualized and designed the study outline, acquired data, and supervised the analysis of data; Jae Ho Cho wrote and revised the manuscript; Hyuk Yoon and Young Soo Park collected the data, and revised the manuscript; and Cheol Min Shin and Nayoung Kim analyzed and interpreted the data. All authors have reviewed and approved the final manuscript.

**Conceptualization:** Dong Ho Lee.

**Data curation:** Hyuk Yoon, Young Soo Park.

**Formal analysis:** Hyuk Yoon, Cheol Min Shin, Young Soo Park, Nayoung Kim.

**Methodology:** Cheol Min Shin.

**Project administration:** Dong Ho Lee.

**Supervision:** Dong Ho Lee.

**Writing – original draft:** Jae Ho Cho.

**Writing – review & editing:** Jae Ho Cho, Cheol Min Shin.
